# Impact of COVID‐19 on carers of people with dementia in the community: Findings from the British IDEAL cohort

**DOI:** 10.1002/gps.5708

**Published:** 2022-04-08

**Authors:** Catherine Quinn, Laura D. Gamble, Sophie Parker, Anthony Martyr, Rachel Collins, Christina Victor, Eleanor Dawson, Anna Hunt, Claire Pentecost, Louise Allan, Linda Clare

**Affiliations:** ^1^ Centre for Applied Dementia Studies, Faculty of Health Studies Bradford University Bradford UK; ^2^ Wolfson Centre for Applied Health Research Bradford UK; ^3^ Population Health Sciences Institute Newcastle University Newcastle upon Tyne UK; ^4^ College of Medicine and Health University of Exeter Exeter UK; ^5^ College of Health, Medicine and Life Sciences Brunel University London London UK; ^6^ NIHR Applied Research Collaboration South‐West Peninsula Exeter UK

**Keywords:** caregiver, coping, life satisfaction, stress, support, well‐being

## Abstract

**Objective:**

Unpaid carers for people with dementia play a crucial role in society. Emerging evidence suggests the COVID‐19 pandemic has negatively impacted on carers. This study sought to explore the impact of the COVID‐19 pandemic on carers for community‐dwelling people with dementia and compare responses with pre‐pandemic data.

**Methods:**

Data were collected between September 2020 and April 2021 in England and Wales. Carers were identified from the Improving the experience of Dementia and Enhancing Active Life (IDEAL) cohort and data were collected either through the telephone, video conferencing, or an online questionnaire. Responses from 242 carers were compared against benchmark data from the IDEAL cohort collected pre‐pandemic. Analyses were conducted for the full sample of carers and spousal/partner carers only.

**Results:**

In total 48.8% of carers thought their healthcare needs were negatively affected during the pandemic. Compared with pre‐pandemic data carers were more lonely and experienced less life satisfaction. There was little impact on carers' experience of caregiving, although carers felt trapped in their caregiving role. Carers were more optimistic and had higher social contact with relatives. There were changes in the methods carers used for contacting relatives and friends. Most carers coped very or fairly well during the pandemic. There was little difference in the experiences of spousal/partner carers and the full sample.

**Conclusions:**

After a long period of providing care under pandemic conditions carers require additional support. This support needs to be focused on alleviating feelings of loneliness and increasing life satisfaction. Services need to consider how to improve access to health care, particularly resuming face‐to‐face appointments.

## Key points

- Carers experienced challenges in accessing health care, were more lonely, and experienced less life satisfaction.
- Carers felt they were coping well, were more optimistic, and had higher social contact with relatives.
- Carers need to be appropriately supported to ensure they are as satisfied as possible with their lives and have opportunities for social contact outside the caring role.
- Healthcare services need to consider how to improve and maintain access to health care under pandemic conditions.

Unpaid carers, often family members or friends, have a crucial role in supporting people with dementia. Unpaid care accounts for 40% of the costs of dementia in the United Kingdom.^1^ Those caring for people with dementia are often giving extensive assistance over a long period of time.^2^ Multiple factors can influence the capability of carers to live well, such as psychological health, physical fitness and health, and their experiences of caregiving.^3^ The carer's well‐being can also influence the well‐being of the person with dementia.^4^ Although carers can identify positive experiences in providing care,^5^, ^6^ it is clear that caregiving can take a considerable toll on the health and well‐being of carers^2^, ^7^, ^8^ and they need appropriate support.^9^

The COVID‐19 pandemic has negatively impacted on people with dementia and carers. Between March and June 2020 in England and Wales, 27.5% of people who died from COVID‐19 had dementia and the largest increase in non‐COVID‐19 deaths was in people with dementia.^10^ Within the UK, periods of national and local lockdowns and periods of restrictions meant that carers had fewer opportunities for respite and experienced reduced access to health and social care.^10^, ^11^, ^12^ There is emerging evidence of the impact of the pandemic on carers. Qualitative studies have highlighted that for carers, the pandemic exacerbated an already difficult situation having a negative impact on well‐being.^11^, ^13^, ^14^, ^15^, ^16^ Given the unprecedented situation and the speed in which data were collected, some quantitative studies just focused on reporting data collected during the pandemic; for example, rates of anxiety, depression, and stress in carers during periods of lockdown/confinement.^17^, ^18^, ^19^ Although these data provide a helpful description of carers' well‐being, without details of the pre‐COVID context, these studies cannot explore the wider impact of the pandemic and resulting restrictions on carers. Retrospective accounts, where carers compare their current situation to a time before the pandemic started^20^, ^21^, ^22^, ^23^, ^24^, ^25^, ^26^, ^27^, ^28^, ^29^, ^30^, ^31^, ^32^ suggest that some carers described increased care needs of the person with dementia,^25^, ^27^, ^29^ self‐reported stress,^21^, ^24^ burden,^22^, ^33^ loneliness,^23^ anxiety and depression,^23^, ^26^, ^32^ although a worsening of well‐being was not universal for all carers taking part in these studies.

Whilst retrospective accounts rely on carers accurately recalling the situation pre‐pandemic, other studies have compared data collected at different timepoints during the pandemic. Most studies collected data during the pandemic outbreak, typically comparing data pre lockdown/confinement and during lockdown/confinement.^34^, ^35^, ^36^, ^37^ In terms of pre‐pandemic, one study states baseline data was collected pre‐pandemic but it's not clear when.^38^ Only one study clearly states baseline data was collected pre‐pandemic (4 months before first infected case).^39^ The findings from all these studies indicate increases in carer stress,^35^ burden,^34^, ^39^ and worsening of well‐being^39^ during lockdown. Although, other studies reported no changes in carer health‐related quality of life^36^ or distress.^38^ A study of caregivers of people with subcortical vascular data that collected data over three timepoints found that although anxiety, depression, and stress increased when assessed at the end of a lockdown period, these levels had started to decrease 2 months later.^37^ These findings suggest that things may change over time as carers adjust to the situation. Whilst these studies start to provide an important picture of changes in carers' circumstances, due to the nature of data collection, these studies typically relied on audits of health records and samples of convenience.

To date there is no study of carers of people with dementia that has compared the current impact of the pandemic with equivalent benchmarked pre‐pandemic information from carers. As identified most studies collected data during the pandemic. By using equivalent pre‐pandemic information this would provide important insights into changes experienced by carers during the pandemic. The aim of this study was to explore the impact of the pandemic on carers of people with dementia residing in the community who were already participating in the Improving the experience of Dementia and Enhancing Active Life (IDEAL) cohort,^40^, ^41^ thus allowing comparison of data collected during the second wave of the pandemic with pre‐pandemic data.

## 1 METHOD

### 1.1 Design

This study was cross‐sectional where data collected from carers of community‐dwelling people with dementia during the COVID‐19 pandemic were compared against benchmarked data collected before the pandemic as part of IDEAL.

INCLUDE (Identifying and mitigating the individual and dyadic impact of COVID‐19 and life under physical distancing on people with dementia and carers) focussed specifically on the impact of the pandemic on people with dementia and carers.^42^ Data were collected between 21 September 2020 and 30 April 2021 in participants living in England and Wales. This was a period of continuing and changing restrictions, with local and national lockdowns, and the commencement of the vaccination programme in December 2020.

The participants for INCLUDE were identified from a pre‐existing cohort study called the IDEAL programme. IDEAL recruited people with dementia and carers to take part in the study. Participants were followed up over a number of timepoints. At Time 1 (T1) there were 1537 community‐dwelling people with mild‐to‐moderate dementia and 1277 carers recruited from 29 National Health Service sites within England, Scotland, and Wales between June 2014 and August 2016. The cohort was followed up yearly for two further timepoints. A further study, IDEAL‐2, would have followed the cohort for three additional timepoints (T4‐ T6). Data collection for T4 and T4, however, were affected by the pandemic. As part of IDEAL‐2, an enrichment cohort was recruited to increase the number of people with rarer dementias, young onset dementia, and people over 90. Participants recruited into INCLUDE were identified from the existing IDEAL cohort and from this enrichment cohort.^42^

To explore the effect of the pandemic we compared data collected from carers taking part in the INCLUDE study against pre‐pandemic data (referred to as benchmarked data). For the benchmarked data we used data collected from IDEAL T3, the most recent complete dataset, collected from 706 carers of people with dementia residing in the community between 2016 and 2018. This study focuses on carers of community‐dwelling people with dementia, thus carers of people living in care were excluded from analyses.

IDEAL was approved by Wales Research Ethics Committee 5 (reference 13/WA/0405) and IDEAL‐2 by Wales Research Ethics Committee 5 (reference 18/WS/0111) and Scotland A Research Ethics Committee (reference 18/SS/0037). INCLUDE was approved as an amendment to IDEAL‐2 for England and Wales (18/WS/0111 AM12). IDEAL and IDEAL‐2 are registered with the UK Clinical Research Network (UKCRN), numbers 16593 and 37,955.

### 1.2 Participants

Participants were eligible to take part in INCLUDE if they were living in England or Wales and had previously participated in IDEAL. Carers could participate if the person with dementia was not taking part.

### 1.3 Measures

The structured interview drew on the domains of living well explored previously,^41^ incorporating single items used in IDEAL and additional questions focusing on experiences during the pandemic. The questions covered health, social networks, psychological well‐being, and caregiving experiences. A full description of the measures is provided in Supplementary Tables 2–5.

### 1.4 Procedure

Data were collected by three trained interviewers who were graduate or masters level psychologists. Potential participants were contacted either through letter, telephone, or email. During this initial contact, interviewers provided information about the study and answered questions. A follow‐up call was arranged to take informed consent. Carers could either be interviewed over the telephone, video conferencing, or self‐complete the questionnaire online.

### 1.5 Data analyses

Questions on experiences during the pandemic were analysed descriptively. The other items were compared against IDEAL T3 data. Version 5 of the IDEAL datasets was used. Depending on whether the data were categorical or ordinal, chi‐square or Mann‐Whitney U tests were used to compare the difference in responses prior to and during the COVID‐19 pandemic. As INCLUDE participants comprised people from both the main IDEAL cohort and the enrichment cohort, in relation to the T3 data INCLUDE responses contained a mixture of paired and independent responses. Therefore, sensitivity analyses were conducted using the ‘PartiallyOverlapping’ package in R which compares proportions using the partially overlapping samples *z*‐test (for categorical variables) and means through the partially overlapping *t*‐test (for continuous and ordinal variables). Differences in the paired samples were additionally explored using the Friedman and McNemar's chi‐squared tests.

Analyses were conducted for the full sample of carers. Since most of the carers were spouses/partners, we explored this specific group to determine if there were any differences in responses.

## 2 RESULTS

**FIGURE 1:**
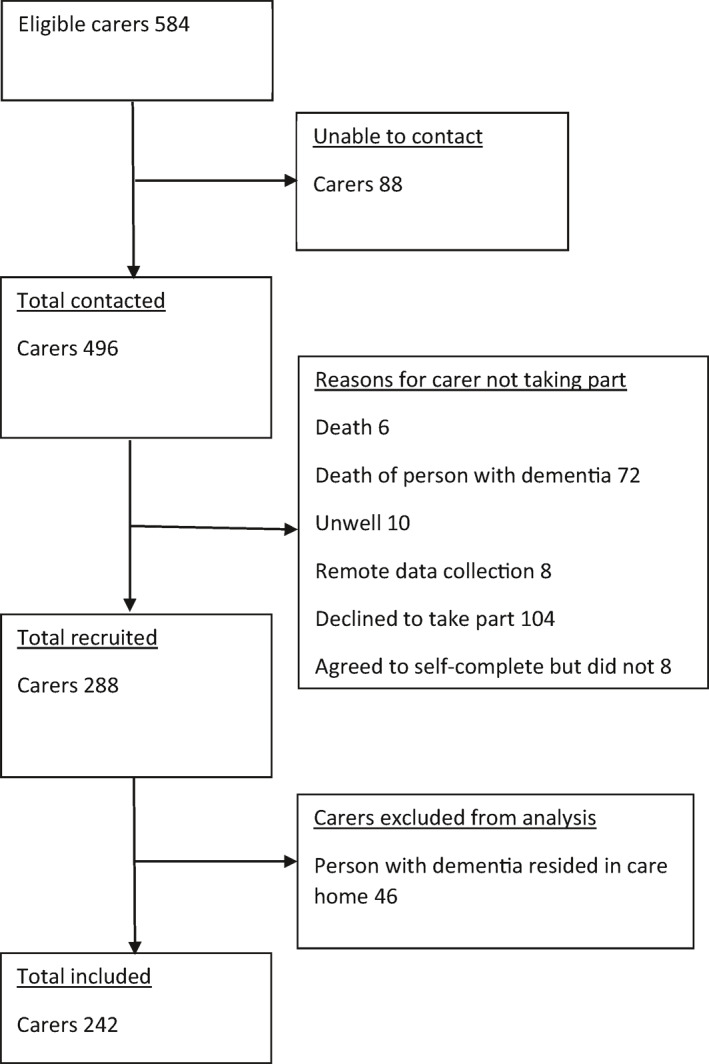
Flowchart summarising the recruitment process and reasons for withdrawal

A flowchart of the recruitment process and reasons for withdrawal is provided in Figure 1. Out of the 584 eligible carers, researchers were able to contact 496. Of these 288 agreed to participate; 46 carers were excluded from the current study as the person with dementia was in residential care yielding a sample of 242 carers. Participants are described in Table 1; most carers were female (68.2%) and spouse/partners (85.95%). Participants of IDEAL T3 (benchmarked‐dataset) are described in Supplementary Table 1.

**TABLE 1:** Characteristics of the carers of people with dementia

		Entire cohort	Spouses/partners only
			*N* (%)
		*N* (%)	
Carer sex	Female	165 (68.2)	138 (66.3)
Carer age	<65	58 (24.0)	37 (17.8)
	65–69	45 (18.6)	36 (17.3)
	70–74	57 (23.6)	55 (26.4)
	75–79	31 (12.8)	31 (14.9)
	80+	51 (21.1)	49 (23.6)
Carer ethnicity	White British	226 (93.4)	194 (93.3)
	White other	7 (2.9)	7 (3.4)
	Other	3 (1.2)	3 (1.4)
	Missing	6 (2.5)	4 (1.9)
Carer education	No qualifications	33 (13.6)	31 (14.9)
	School leaving certificate at age 16	55 (22.7)	50 (24.0)
	School leaving certificate at age 18	66 (27.3)	54 (26.0)
	University	74 (30.6)	63 (30.3)
	Missing	14 (5.8)	10 (4.8)
Carer marital status	Single	6 (2.5)	
	Married; first	156 (64.5)	143 (68.8)
	Remarried	50 (20.7)	49 (23.6)
	Civil partnership	2 (0.8)	2 (1.0)
	Legally separated	2 (0.8)	
	Divorced	12 (5.0)	5 (2.4)
	Widowed	1 (0.4)	
	Cohabiting	12 (5.0)	9 (4.3)
	Missing	1 (0.4)	
Carer relationship	Spouse/partner	208 (85.9)	208 (100.0)
	Other family/friend	34 (14.1)	0 (0)
Person with dementia sex	Female	94 (38.8)	60 (31.6)
Person with dementia age	<65	29 (12.0)	25 (12.0)
	65–69	36 (14.9)	36 (17.3)
	70–74	38 (15.7)	33 (15.9)
	75–79	45 (18.6)	43 (20.7)
	80+	94 (38.8)	71 (34.1)
Person with dementia diagnosis	Alzheimer's disease (AD)	114 (47.1)	103 (59.5)
	Vascular dementia (VaD)	22 (9.1)	17 (8.2)
	Mixed AD and VaD	35 (14.5)	23 (11.1)
	Frontotemporal dementia	32 (13.2)	29 (13.9)
	Parkinson's disease dementia	11 (4.5)	10 (4.8)
	Dementia with Lewy bodies	20 (8.3)	19 (9.1)
	Unspecified/Other	8 (3.3)	7 (3.4)

### 2.1 Health and health care

Reponses are provided in Table 2 and Supplementary Table 2 For the full sample (both spouse/partners and family/friends) a small percentage had contracted COVID‐19, which was lower than population norms (4.1% vs. 6.1%). In total 21.1% reported knowing someone close to them who had contracted COVID‐19. The pandemic impacted on carers' health with 48.8% reporting their healthcare needs were affected. This related to cancelled or postponed hospital appointments, operations, and medical reviews. There were difficulties getting appointments with general practitioners, opticians, and dentists, particularly face‐to‐face appointments. Only 24% avoided seeking help for health issues, primarily due to not wanting to burden services, feeling the issue was not urgent, fear of contracting COVID‐19 in medical settings, and dislike of telephone appointments. Analyses on spouse/partner carers only yielded similar findings.

**TABLE 2:** Experiences of health and healthcare

	Benchmark for comparison where applicable^a^	All carers *n* = 242	Spouse/partners only *n* = 208
Direct experience of COVID‐19:			
Had COVID‐19	Population^b^	4.1% (6.1%)	3.8% (6.1%)
Treated in hospital for COVID‐19		0.4%	0.5%
Someone close to you had COVID‐19		21.1%	20.2%
Health during the pandemic:			
Overall health poor or very poor	IDEAL T3	9.6% (8.0%)	10.6% (8.1%)
Healthcare needs affected by pandemic		48.8%	48.6%
Healthcare services stopped due to pandemic		21.1%	19.2%

^a^ Benchmark data are shown in brackets after the equivalent data for the current sample.

^b^ Population estimate for people aged 50+ in England as of 8th May 2021; *source*: https://coronavirus.data.gov.uk/details/download.

### 2.2 Social network

Responses are provided in Table 3 and Supplementary Table 3. For the full sample, there was a significant increase in the number of relatives the carer was in contact with at least monthly compared to IDEAL T3 (6.2 vs. 5.3) which was also supported by the sensitivity analyses (Supplementary Table 6). When asked, 45.9% of carers said this contact had changed since the pandemic. Responses indicate changes related to less face‐to‐face contact and an increased use of telephone/virtual contact.

**TABLE 3:** Contact with family and friends

	Benchmark for comparison where applicable^b^	All carers^a^ *n* = 242	Spouse/partners only^a^ *n* = 208
Number of relatives in contact at least monthly	IDEAL T3	**6.2 ± 3.9 (5.3 ± 4.4)**	**6.1 ± 3.9 (5.3 ± 4.6)**
Has this changed since the coronavirus outbreak? Yes		45.9%	45.2%
Very/slightly satisfied with support from family	IDEAL T3	77.2% (68.8%)	78.3% (69.9%)
Number of friends in contact at least monthly	IDEAL T3	5.3 ± 4.9 (5.5 ± 4.7)	5.4 ± 5.1 (5.6 ± 4.7)
Has this changed since the coronavirus outbreak? Yes		56.2%	56.7%
Very/slightly satisfied with support from friends	IDEAL T3	73.6% (62.6%)	72.6% (61.2%)

^a^ Bold type indicates a statistically significant difference at the 5% level.

^b^ Benchmark data are shown in brackets after the equivalent data for the current sample.

Although not significant, carers reported more satisfaction with support from relatives (77.2% vs. 68.8%). The number of friends in contact at least monthly was not significantly different, though 56.2% of carers said this had changed since the pandemic for similar reasons as above. The increase in satisfaction with support from friends (73.6% vs. 62.6%) was not significant. Similar findings were observed for spouse/partner carers only.

### 2.3 Psychological well‐being and quality of life

For the full sample, significantly more carers reported feeling lonely than at IDEAL T3 (54.5% vs. 28.7%); they also reported slightly less life satisfaction (6.1 vs. 6.6), see Table 4 and Supplementary Table 4. These findings were supported by the sensitivity analyses (Supplementary Table 6). Carers were more optimistic, expecting more good things to happen than bad (56.7% vs. 53.4%), and this was supported by the paired samples tests (Supplementary Table 6). Other measures of well‐being were not significantly different. Findings for spouse/partners only were similar although fewer spouses/partners reported having a good or very good quality of life (54.3% vs. 58.2%), a difference which was lost when combining them with family/friend carers. However, this was not supported in the sensitivity analysis (Supplementary Table 7).

**TABLE 4:** Psychological well‐being and quality of life

	Benchmark for comparison where applicable^b^	All carers^a^ *n* = 242	Spouse/partners only^a^ *n* = 208
Feel lonely (yes, more or less)	IDEAL T3	**54.5% (28.7%)**	**52.9% (29.9%)**
Cheerful & in good spirits >50% of time last 2 weeks	IDEAL T3	66.9% (68.0%)	66.7% (68.0%)
Expect more good things to happen than bad	IDEAL T3	**56.7% (53.4%)**	**54.8% (53.3%)**
Satisfied with life (0–10 scale)	IDEAL T3	**6.1 ± 2.2 (6.6 ± 2.1)**	**6.1 ± 2.2 (6.5 ± 2.1)**
Feel the things I do are worthwhile (0–10)	IDEAL T3	7.2 ± 2.1 (7.5 ± 1.9)	7.2 ± 2.2 (7.4 ± 1.9)
Good or very good quality of life	IDEAL T3	57.8% (60.9%)	**54.3% (58.2%)**
Coped very or fairly well during the pandemic		94.7%	95.7%
Pandemic had positive aspects or benefits		55.8%	54.8%
Fairly or very easy to keep oneself occupied during the pandemic		85.1%	85.1%

^a^ Bold type indicates a statistically significant difference at the 5% level.

^b^ Benchmark data are shown in brackets after the equivalent data for the current sample.

Most carers felt that they had coped very or fairly well during the pandemic (94.7%) and around half identified that the pandemic had positive aspects (55.8%). Most carers found it easy to keep themselves occupied (85.1%). Findings were similar for spouse/partner carers only.

### 2.4 Carers' experience of caregiving

Compared with IDEAL T3 more carers felt trapped by the person's dementia (42.2% vs. 37.1%). However, this finding was not supported in the sensitivity analyses (Supplementary Table 6). In terms of support available, carers were more likely to identify someone to step in to help generally (50% vs. 35.4%) and if the carer needed a break (40.9% vs. 28.2%). These findings were supported by the sensitivity analyses. Findings were similar when looking at spouse/partner carers only, see Table 5 and Supplementary Table 5.

**TABLE 5:** Carers' experience of caregiving

	Benchmark for comparison where applicable^b^	All carers^a^ *n* = 242	Spouse/partners only^a^ *n* = 208
Competence:			
Meeting needs of person with dementia (most/all of the time)	IDEAL T3	82.2% (80.3%)	82.7% (81.2%)
Doing a good job as a carer (most/all of the time)	IDEAL T3	75.2% (77.0%)	75.0% (78.3%)
Feel competent in ability to care (most/all of the time)	IDEAL T3	83.5% (80.5%)	84.6% (81.1%)
Relationship with person with dementia:			
Get along (well, very, extremely)	IDEAL T3	83.9% (82.2%)	82.7% (81.5%)
Coping:			
Cope as a carer (often/always)	IDEAL T3	70.3% (63.8%)	69.8% (64.6%)
Social restrictions:			
If ill, is there someone to step in to help (yes easily)	IDEAL T3	**50.0% (35.4%)**	**47.6% (35.2%)**
If needed a break, is there someone to step in to help (yes easily)	IDEAL T3	**40.9% (28.2%)**	**38.9% (26.9%)**
Role captivity:			
Do you wish you were free to lead a life of your own (somewhat/very much)	IDEAL T3	36.4% (33.0%)	36.1% (31.5%)
Do you feel trapped by person's dementia (somewhat/very much)	IDEAL T3	**42.2% (37.1%)**	**43.3% (37.4%)**
Do you wish you could just run away (somewhat/very much)	IDEAL T3	23.9% (17.6%)	22.6% (16.9%)

^a^ Bold type indicates a statistically significant difference at the 5% level.

^b^ Benchmark data are shown in brackets after the equivalent data for the current sample.

Other care‐related questions did not show any significant differences, with similar findings in all carers and spouses/partners only. There was no difference in the quality of the dyadic relationship. More carers reported improvements in their ability to cope with caregiving (70.3% vs. 63.8%), ability to meet the person's needs (82.2% vs. 80.3%), and competency in their ability to care (83.5% vs. 80.5%). There was little difference in the proportion saying that they got along with the person with dementia (83.9% vs. 82.2%). Slightly fewer reported feeling they were doing a good job as a carer (75.2% vs. 77%). More reported wishing to be free to lead a life of their own (36.4% vs. 33%) and wishing they could run away (23.9% vs. 17.6%).

## 3 DISCUSSION

To the best of our knowledge this is the first study to compare the impact of the COVID‐19 pandemic on carers of people with dementia with equivalent pre‐pandemic data. The current study involved carers from the IDEAL cohort, providing a unique insight into the effect of the pandemic on carers. Overall, findings were equivocal indicating some impact on carers' health, social contact, well‐being, and experience of caregiving. There was little difference when considering the experiences of spouses/partners only versus the full sample.

Although there was no difference in carers' overall health, just under half said their healthcare needs were affected by the pandemic with difficulties accessing medical appointments and treatments. This may reflect wider issues relating to access to healthcare for people with dementia and carers^10^ and the move to more remote consultations. Difficulties in accessing healthcare during the pandemic has been reported by other groups, such as older people with long‐term health conditions.^43^ Approximately half of carers identified changes in contact with relatives and friends, and there was increased contact with relatives compared with IDEAL T3. The findings indicate carers have used telephone/technology to connect with others during the pandemic, similar to other findings.^16^, ^21^ Despite this increased contact, carers were significantly lonelier when compared to IDEAL T3, which aligns with findings from other studies.^11^, ^23^ This finding may reflect a lack of relationship closeness, particularly if the main source of contact is the person with dementia. There may have been fewer opportunities to have meaningful face‐to‐face contact with others outside the household. Similar reports of increased loneliness during the pandemic have been identified in older people.^43^ In a survey of 5904 carers just under half of carers reported feeling lonely and cut off.^44^

Other well‐being findings were equivocal. Carers had lower life satisfaction than at T3. Other studies have not yet explored life satisfaction in carers during the pandemic. The pandemic would have brought about many changes to the lives of carers and this potentially could have impacted on their overall life satisfaction. There is also evidence of a link between low life satisfaction and loneliness.^45^ Interestingly, carers were more optimistic, and similar findings have been reported in people with dementia.^42^ Increased optimism may be a form of coping, concordant with theories that positive and negative psychological states can co‐occur during stressful circumstances^46^ and finding meaning in adversity can be a form of coping.^5^, ^47^ Certainly, just over half of the carers identified that the pandemic had some positives and most carers reported coping well during the pandemic. Although studies have reported carers struggling to cope during the pandemic^12^ others have reported carers coping well^26^ utilising active and passive coping strategies.^18^ Our findings may reflect carers' adaptation to the situation.

There was very little impact on the carers' experience of caregiving. Compared with IDEAL T3 more carers felt trapped by the person's dementia. It is possible that with people with dementia being identified as more clinically vulnerable to COVID‐19^48^ this placed additional pressure on carers to keep them safe. More carers were able to identify someone that could provide help than at IDEAL T3. This finding may seem contradictory with carers reporting increased caregiving responsibilities^11^, ^27^, ^39^ and a lack of access to formal support services.^12^ However, it may reflect that most carers had increased contact with family and thus a larger support network. Equally it may reflect the data collection period which started 9 months into the pandemic by which time some support services had restarted, albeit often through remote delivery.

It is important to recognise limitations of the study. Data collection was through the use of single items from validated measures, limiting the range of data collected. However, this approach was to reduce burden on carers and allow comparison with existing IDEAL data. For the analyses, data from INCLUDE participants were compared with IDEAL T3 data, collected between 2016 and 2018, the last complete timepoint. Thus, it is important to acknowledge this gap when comparing findings with benchmarked data. The differences observed may not necessarily be solely attributable to the pandemic; however, they do provide an indication of what might have changed. This study excluded carers of people with dementia living in care homes. The rationale for this was that it was envisaged that the experiences of carers for people with dementia in care homes would be very different from those living in the community and so this data needed to be analysed separately. The sample size for carers of people with dementia in care homes in the INCLUDE dataset was too small to undertake analyses as a separate sub‐group for this paper; however, their experiences will be reported in other papers from this dataset.

Both the existing IDEAL and enrichment cohorts were used to identify participants for the current study, and while the participants were similar the enrichment cohort included a higher proportion of younger carers (<65). The carers who participated in INCLUDE may have been coping better than those who declined to take part. However, unpublished qualitative interviews with INCLUDE carers illustrate the challenges they faced and align with our reflection that they have had little choice but to cope. The statistical tests employed assumed the INCLUDE and IDEAL T3 samples were independent of each other, but they were partially overlapping and incorporated both paired and unpaired samples. Therefore, sensitivity analyses were conducted using the partially overlapping *t*‐test or *z*‐test, and paired samples tests on paired data only. The partially overlapping tests were not able to handle categorical variables but since the categorical variables were ordered they were treated as ordinal and *t*‐tests were conducted.^49^ Therefore, findings need to be interpreted with caution when the sensitivity analyses do not concur with the results of the main analyses.

The study provides some important insights into the experiences of carers during the pandemic, exploring domains not previously reported in other studies. The quantitative methods employed allowed for comparison with pre‐pandemic data. However, additional research employing a qualitative methodology could further explore some of the findings of this study in the context of wider social/cultural factors. For example, in relation to coping using qualitative methodology O’Rourke et al.^15^ were able to categorise carers and people with dementia into three groups through their ability to cope with the pandemic. Participants who were ‘just coping’ spoke of increasing tension in their relationship and lack of access to an outdoor space. Qualitative interviews have been undertaken with carers from the INCLUDE sample at three time‐points during the pandemic and these are expected to provide additional insights into the carers' experiences.

Some recommendations for health and social care professionals can be made based on the findings of this study. Given that carers reported their healthcare needs had been affected it is important to consider how access to healthcare and healthcare delivery can be improved. Further research is needed to explore the long‐term impact of the pandemic on access to health care, particularly with the increased use of telemedicine.^50^ Some carers in this study had a clear preference for face‐to‐face appointments and had declined remote consultations or had found it difficult to discuss issues over the telephone. Therefore, it is important that there are options for face‐to‐face appointments to enable people to access services. Another issue to address is loneliness. Loneliness in carers was already an issue pre‐pandemic^45^; therefore, better support is needed to increase opportunities for social contact and to enable individuals to develop good quality relationships with others. This may be through more opportunities for carers to meet with other carers or more broadly others within their community. Having local community centres or befriending schemes have been identified by carers as a way of increasing social contact.^51^ Carers had lower life satisfaction indicating the need for greater investment in supporting carers in their role.^52^ Although the findings indicate that generally carers coped well, it may be that carers had little choice but to adjust to and cope with the situation. The long‐term effects of this are unknown and there are potential risks of burnout. Health and social care professionals need to consider that carers may require additional support in light of this long period of providing care under pandemic conditions. Carers have played a vital role within this pandemic and they need to be appropriately supported to enable them to continue within their caregiving role.

## CONFLICT OF INTEREST

The authors have no conflict of interest to report.

## AUTHOR CONTRIBUTIONS

Conception and design – Catherine Quinn, Linda Clare, Anthony Martyr, Claire Pentecost, Rachel Collins, Louise Allan, Christina Victor. Analysis and interpretation of data Catherine Quinn, Laura D. Gamble, Sophie Parker. Drafting the manuscript – Catherine Quinn. Critical appraisal and review of the manuscript – Laura D. Gamble, Sophie Parker, Anthony Martyr, Claire Pentecost, Rachel Collins, Eleanor Dawson, Anna Hunt, Louise Allan, Christina Victor, Linda Clare. Final approval of the manuscript – all.

## Supporting information

Supporting Information

## Data Availability

IDEAL data were deposited with the UK data archive in April 2020 and will be available to access from April 2023. Details of how the data can be accessed after that date can be found here: https://reshare.ukdataservice.ac.uk/854293
