# Supplementary material for: Impact of COVID‐19 on carers of people with dementia in the community: Findings from the British IDEAL cohort
Source: Int J Geriatr Psychiatry. 2022 Apr 8;37(5):10.1002/gps.5708. doi: 10.1002/gps.5708 (PMC9087398; doi:10.1002/gps.5708)
Supplement: Supplementary file 1 — Supporting Information [file GPS-37--s001.docx]

Supplementary Table 1. IDEAL T3 carer dataset characteristics

|  |  | All carers (n= 706): N (%) or mean (SD); range | Spouse/partners only (N=603): N (%) or mean (SD); range |
| --- | --- | --- | --- |
| Carer Sex | Female | 481 (68.1) | 393 (65.2) |
| Carer Age | <65 | 154 (21.8) | 66 (10.9) |
|  | 65-69 | 113 (16.0) | 104 (17.2) |
|  | 70-74 | 162 (22.9) | 160 (26.5) |
|  | 75-79 | 125 (17.7) | 123 (20.4) |
|  | 80+ | 152 (21.5) | 150 (24.9) |
|  | Mean (SD); range | 71.1 (10.5); 28-94 | 73.7 (8.0); 43-94 |
| Carer ethnicity | White British | 663 (93.9) | 571 (94.7) |
|  | White Other | 18 (2.5) | 17 (2.8) |
|  | Other | 5 (0.7) | 4 (0.7) |
|  | Missing | 20 (2.8) | 11 (1.8) |
| Carer education | No qualifications | 144 (20.4) | 138 (22.9) |
|  | school leaving certificate at age 16 | 156 (22.1) | 138 (22.9) |
|  | school leaving certificate at age 18 | 206 (29.2) | 171 (28.4) |
|  | University | 189 (26.8) | 149 (24.7) |
|  | Missing | 11 (1.6) | 7 (1.2) |
| Carer marital status | Single | 14 (2.0) | - |
|  | Married; first | 513 (72.7) | 459 (76.1) |
|  | Remarried | 132 (18.7) | 120 (19.9) |
|  | A civil partnership | 5 (0.7) | 4 (0.7) |
|  | Legally separated | 3 (0.4) | 1 (0.2) |
|  | Divorced | 14 (2.0) | 3 (0.5) |
|  | Widowed | 3 (0.4) | 2 (0.3) |
|  | Cohabiting | 22 (3.1) | 14 (2.3) |
| Carer relationship | Spouse/partner | 603 (85.4) |  |
|  | Other family/friend | 103 (14.6) |  |
| Person with dementia Sex | Female | 289 (40.9) | 214 (35.5) |
| Person with dementia Age | <65 | 68 (9.6) | 47 (7.8) |
|  | 65-69 | 108 (15.3) | 56 (9.3) |
|  | 70-74 | 141 (20.0) | 131 (21.7) |
|  | 75-79 | 164 (23.2) | 124 (20.6) |
|  | 80+ | 225 (31.9) | 216 (35.8) |
|  | Mean (SD); range | 74.9 (8.2); 48-95 | 76.2 (7.7); 50-92 |
| Person with dementia diagnosis* | Alzheimer’s disease (AD) | 409 (57.9%) | 348 (57.7%) |
|  | Vascular dementia (VaD) | 65 (9.2%) | 56 (9.3%) |
|  | Mixed AD and VaD | 147 (20.8%) | 119 (19.7%) |
|  | Frontotemporal dementia | 29 (4.1%) | 28 (4.6%) |
|  | Parkinson’s disease dementia | 18 (2.5%) | 17 (2.8%) |
|  | Dementia with Lewy bodies | 22 (3.1%) | 20 (3.3%) |
|  | Unspecified/Other | 16 (2.2%) | 15 (2.5%) |

*Diagnostic information was not available for 38 people with dementia at T3 for all carers, and 29 for the spouse/partners only. For these, their last known diagnosis from an earlier timepoint was reported.

Supplementary Table 2. Experiences of health and healthcare during the pandemic

| Item | Response key | Participants | | Benchmark (where applicable) | Benchmarking data (where applicable) | | | Statistical comparison  (where applicable) | |
| --- | --- | --- | --- | --- | --- | --- | --- | --- | --- |
| Item |  | INCLUDE all carers  N(%) or mean(SD) | INCLUDE spouse/partners only N(%) or mean(SD) |  | Population data | IDEAL T3 all carers N(%) or mean(SD) | IDEAL T3 spouse/partners only N(%) or mean(SD) | INCLUDE all carers vs benchmark | INCLUDE spouse/partners only vs benchmark |
| **Direct experience of COVID-19:** |  |  |  |  |  |  |  |  |  |
| Have you had COVID-19?* | Yes  No | 10 (4.1%)  232 (95.9%) | 8 (3.8%)  200 (96.2%) | Population data for 50+ in England as of 8/5/2021* | 1284225 (6.1%)  19759438 (93.9%) |  |  |  |  |
| Were you treated in hospital for COVID-19? | Yes  No/No prev Q  Missing | 1 (0.4%)  241 (99.6%)  - | 1 (0.5%)  207 (99.5%)  - |  |  |  |  |  |  |
| Has anyone close to you had COVID-19? | Yes  No | 51 (21.1%)  191 (78.9%) | 42 (20.2%)  166 (79.8%) |  |  |  |  |  |  |
| **Health during the pandemic:** |  |  |  |  |  |  |  |  |  |
| Overall, how would you rate your health in the past 4 weeks? ^1^ | Very poor  Poor  Fair  Good  Very good  Excellent  Missing | 4 (1.7%)  19 (7.9%)  62 (25.6%)  94 (38.8%)  47 (19.4%)  16 (6.6%)  - | 4 (1.9%)  18 (8.7%)  56 (26.9%)  77 (37.0%)  42 (20.2%)  11 (5.3%)  - | IDEAL T3 |  | 6 (0.8%)  51 (7.2%)  202 (28.6%)  228 (32.3%)  159 (22.5%)  40 (5.7%)  20 (2.8%) | 5 (0.8%)  44 (7.3%)  183 (30.3%)  192 (31.8%)  139 (23.1%)  21 (3.5%)  19 (3.5%) | *X*^2^ (5) = 4.91, p = 0.428 | *X*^2^ (5) = 5.51, p = 0.357 |
| Have you avoided seeking help for any health issues because of the coronavirus? | Yes  No | 58 (24.0%)  184 (76.0%) | 48 (23.1%)  160 (76.9%) |  |  |  |  |  |  |
| Have your healthcare needs been affected by the coronavirus outbreak – for example, appointments postponed or planned operations delayed? | Yes  No | 118 (48.8%)  124 (51.2%) | 101 (48.6%)  107 (51.4%) |  |  |  |  |  |  |
| Did any healthcare services you were already receiving stop due to coronavirus? | Yes  No | 51 (21.1%)  191 (78.9%) | 40 (19.2%)  168 (80.8%) | NA |  |  |  |  |  |

**Population estimate for people aged 50+ in England as of 8^th^ May 2021; source:* [*https://coronavirus.data.gov.uk/details/download*](https://coronavirus.data.gov.uk/details/download)

Supplementary Table 3. Social networks

| Item | Response key | Participants | | Benchmarking data (where applicable) | | Statistical comparison  (where applicable) | |
| --- | --- | --- | --- | --- | --- | --- | --- |
|  |  | INCLUDE all carers  N(%) or mean(SD) | INCLUDE spouse/partners only N(%) or mean(SD) | IDEAL T3 all carers N(%) or mean(SD) | IDEAL T3 spouse/partners only N(%) or mean(SD) | INCLUDE all carers vs benchmark | INCLUDE spouse/partners only vs benchmark |
| **Family and friends:** |  |  |  |  |  |  |  |
| Considering the people to whom you are related either by birth, marriage, or adoption, how many relatives do you see or hear from at least once a month? ^2^ | 0  1  2  3  4  5  6  7  8  9  10  10+  Mean (SD)  Missing | 2 (0.8%)  5 (2.1%)  19 (7.9%)  35 (14.5%)  35 (14.5%)  33 (13.6%)  26 (10.7%)  16 (6.6%)  15 (6.2%)  11 (4.5%)  19 (7.9%)  25 (10.3%)  6.2 (3.9)  1 (0.4%) | 1 (0.5%)  5 (2.4%)  16 (7.7%)  31 (14.9%)  32 (15.4%)  30 (14.4%)  21 (10.1%)  14 (6.7%)  12 (5.8%)  9 (4.3%)  16 (7.7%)  20 (9.6%)  6.1 (3.9)  1 (0.5%) | 12 (1.7%)  27 (3.8%)  100 (14.2%)  95 (13.5%)  119 (16.9%)  82 (11.6%)  70 (9.9%)  40 (5.7%)  48 (6.8%)  12 (1.7%)  45 (6.4%)  29 (4.1%)  5.3 (4.4)  27 (3.8%) | 8 (1.3%)  19 (3.2%)  86 (14.3%)  78 (12.9%)  105 (17.4%)  71 (11.8%)  64 (10.6%)  34 (5.6%)  38 (6.3%)  10 (1.7%)  40 (6.6%)  24 (4.0%)  5.3 (4.6)  26 (4.3%) | U = 95866, z = -3.99, p < 0.001 | U = 68494, z = -3.16, p = 0.001 |
| Has this changed since the coronavirus outbreak? | No  Yes  Missing | 130 (53.7%)  111 (45.9%)  1 (0.4%) | 113 (54.3%)  94 (45.2%)  1 (0.5%) |  |  |  |  |
| How satisfied are you with the support you receive from family? # | Very dissatisfied  Slightly dissatisfied  Neither satisfied or dissatisfied  Slightly satisfied  Very satisfied  Don’t know†  Missing | 8 (3.3%)  17 (7.0%)  27 (11.2%)  40 (16.5%)  147 (60.7%)  2 (0.8%)  1 (0.4%) | 6 (2.9%)  15 (7.2%)  21 (10.1%)  35 (16.8%)  128 (61.5%)  2 (1.0%)  1 (0.5%) | 30 (4.2%)  45 (6.4%)  117 (16.6%)  111 (15.7%)  375 (53.1%)  3 (0.4%)  25 (3.5%) | 21 (3.5%)  34 (5.6%)  101 (16.7%)  86 (14.3%)  335 (55.6%)  2 (0.3%)  24 (4.0%) | *X^2^* (4) = 5.76, p = 0.218 | *X^2^* (4) = 6.77, p = 0.148 |
| Considering all of your friends including those who live in your neighbourhood, how many friends do you see or hear from at least once a month? ^2^ | 0  1  2  3  4  5  6  7  8  9  10  10+  Mean (SD)  Missing | 17 (7.0%)  19 (7.9%)  39 (16.1%)  24 (9.9%)  28 (11.6%)  24 (9.9%)  28 (11.6%)  7 (2.9%)  11 (4.5%)  3 (1.2%)  18 (7.4%)  23 (9.5%)  5.3 (4.9)  1 (0.4%) | 13 (6.3%)  17 (8.2%)  33 (15.9%)  20 (9.6%)  28 (13.5%)  22 (10.6%)  24 (11.5%)  5 (2.4%)  10 (4.8%)  2 (1.0%)  13 (6.3%)  20 (9.6%)  5.4 (5.1)  1 (0.5%) | 36 (5.1%)  39 (5.5%)  86 (12.2%)  96 (13.6%)  95 (13.5%)  64 (9.1%)  65 (9.2%)  34 (4.8%)  50 (7.1%)  11 (1.6%)  61 (8.6%)  43 (6.1%)  5.5 (4.7)  26 (3.7%) | 31 (5.1%)  31 (5.1%)  74 (12.3%)  80 (13.3%)  81 (13.4%)  54 (9.0%)  57 (9.5%)  26 (4.3%)  44 (7.3%)  11 (1.8%)  50 (8.3%)  40 (6.6%)  5.6 (4.7)  24 (4.0%) | U = 78292, z = -1.03, p = 0.301 | U = 56577, z = -1.20, p = 0.230 |
| Has this changed since the coronavirus outbreak? | No  Yes  Missing | 105 (43.4%)  136 (56.2%)  1 (0.4%) | 89 (42.8%)  118 (56.7%)  1 (0.5%) |  |  |  |  |
| How satisfied are you with the support you receive from friends? # | Very dissatisfied  Slightly dissatisfied  Neither satisfied or dissatisfied  Slightly satisfied  Very satisfied  Don’t know†  Missing | 2 (0.8%)  9 (3.7%)  49 (20.2%)  44 (18.2%)  134 (55.4%)  -  4 (1.7%) | 2 (1.0%)  9 (4.3%)  43 (20.7%)  38 (18.3%)  113 (54.3%)  -  3 (1.4%) | 24 (3.4%)  22 (3.1%)  174 (24.6%)  110 (15.6%)  332 (47.0%)  19 (2.7%)  25 (3.5%) | 23 (3.8%)  21 (3.5%)  149 (24.7%)  95 (15.8%)  274 (45.4%)  17 (2.8%)  24 (4.0%) | *X^2^* (4) = 8.74, p = 0.068 | *X^2^* (4) = 7.92, p = 0.095 |

*† treated as missing for statistical tests; #Question taken from IDEAL survey.*

Supplementary Table 4. Psychological well-being and quality of life

| Item | Response key | Participants | | Benchmarking data (where applicable) | | Statistical comparison  (where applicable) | |
| --- | --- | --- | --- | --- | --- | --- | --- |
|  |  | INCLUDE all carers  N(%) or mean(SD) | INCLUDE spouse/ partners only  N(%) or mean(SD) | IDEAL T3 all carers N(%) or mean(SD) | IDEAL T3 spouse/ partners only N(%) or mean(SD) | INCLUDE all carers vs benchmark | INCLUDE spouse/partners only vs benchmark |
| **Negative feelings:**  Do you feel lonely?# | Yes  More or less  No  Missing | 26 (10.7%)  106 (43.8%)  109 (45.0%)  1 (0.4%) | 23 (11.1%)  87 (41.8%)  97 (46.6%)  1 (0.5%) | 87 (12.3%)  116 (16.4%)  474 (67.1%)  29 (4.1%) | 74 (12.3%)  106 (17.6%)  396 (65.7%)  27 (4.5%) | *X^2^* (2) = 55.62, p < 0.001 | *X^2^* (2) = 46.44, p < 0.001 |
| **Positive feelings:** |  |  |  |  |  |  |  |
| In the last two weeks, how much of the time have you felt cheerful and in good spirits? ^3^ | At no time  Some of the time  Less than half the time  More than half the time  Most of the time  All of the time  Missing | 7 (2.9%)  33 (13.6%)  38 (15.7%)  64 (26.4%)  90 (37.2%)  8 (3.3%)  2 (0.8%) | 7 (3.4%)  27 (13.0%)  33 (15.9%)  55 (26.4%)  76 (36.5%)  8 (3.8%)  2 (1.0%) | 4 (0.6%)  79 (11.2%)  116 (16.4%)  197 (27.9%)  254 (36.0%)  29 (4.1%)  27 (3.8%) | 3 (0.5%)  65 (10.8%)  99 (16.4%)  170 (28.2%)  215 (35.7%)  25 (4.1%)  26 (4.3%) | *X^2^* (5) = 9.57, p = 0.088 | *X^2^* (5) = 10.88, p = 0.054 |
| Overall, would you say you expect more good things to happen to you than bad? ^4^ | Strongly disagree  Disagree  Neutral  Agree  Strongly agree  Missing | 5 (2.1%)  43 (17.8%)  56 (23.1%)  95 (39.3%)  42 (17.4%)  1 (0.4%) | 4 (1.9%)  40 (19.2%)  49 (23.6%)  79 (38.0%)  35 (16.8%)  1 (0.5%) | 7 (1.0%)  63 (8.9%)  235 (33.3%)  325 (46.0%)  52 (7.4%)  24 (3.4%) | 5 (0.8%)  55 (9.1%)  200 (33.2%)  279 (46.3%)  42 (7.0%)  22 (3.6%) | *X^2^* (4) = 39.55, p < 0.001 | *X^2^* (4) = 37.32, p < 0.001 |
| **Coping with the pandemic:** |  |  |  |  |  |  |  |
| Have there been any positive aspects or benefits of the coronavirus outbreak? | Yes  No  Missing | 135 (55.8%)  106 (43.8%)  1 (0.4%) | 114 (54.8%)  94 (45.2%) |  |  |  |  |
| Overall how well do you feel you have coped since the coronavirus outbreak? | Not very well  Fairly well  Very well | 13 (5.4%)  142 (58.7%)  87 (36.0%) | 9 (4.3%)  126 (60.6%)  73 (35.1%) |  |  |  |  |
| How easy or difficult was it to keep yourself occupied at home during the coronavirus lockdown? | Not very easy  Fairly easy  Very easy | 36 (14.9%)  114 (47.1%)  92 (38.0%) | 31 (14.9%)  99 (47.6%)  78 (37.5%) |  |  |  |  |
| **Quality of life:** |  |  |  |  |  |  |  |
| On a scale of zero to 10, where zero is “not at all” and 10 is “very”, how satisfied are you with your life nowadays? ^5^ | 0 Not at all  1  2  3  4  5  6  7  8  9  10 Very  Mean (SD)  Missing | 3 (1.2%)  4 (1.7%)  5 (2.1%)  21 (8.7%)  17 (7.0%)  45 (18.6%)  23 (9.5%)  47 (19.4%)  53 (21.9%)  14 (5.8%)  10 (4.1%)  6.1 (2.2)  - | 3 (1.4%)  4 (1.9%)  4 (1.9%)  19 (9.1%)  17 (8.2%)  41 (19.7%)  19 (9.1%)  37 (17.8%)  40 (19.2%)  14 (6.7%)  10 (4.8%)  6.1 (2.2)  - | 10 (1.4%)  11 (1.6%)  -  36 (5.1%)  54 (7.6%)  82 (11.6%)  99 (14.0%)  125 (17.7%)  163 (23.1%)  62 (8.8%)  39 (5.5%)  6.6 (2.1)  25 (3.5%) | 9 (1.5%)  10 (1.7%)  -  34 (5.6%)  47 (7.8%)  66 (10.9%)  89 (14.8%)  105 (17.4%)  137 (22.7%)  50 (8.3%)  33 (5.5%)  6.5 (2.1)  23 (3.8%) | U = 73098, z = -2.65, p < 0.008 | U = 53043, z = -2.61, p < 0.009 |
| On a scale of zero to 10, where zero is “not at all” and 10 is “very”, to what extent do you feel the things you do in your life are worthwhile? ^5^ | 0 Not at all  1  2  3  4  5  6  7  8  9  10 Very  Mean (SD)  Missing | 0 (0.0%)  0 (0.0%)  5 (2.1%)  12 (5.0%)  13 (5.4%)  23 (9.5%)  17 (7.0%)  43 (17.8%)  53 (21.9%)  34 (14.0%)  37 (15.3%)  7.2 (2.1)  5 (2.1%) | 0 (0.0%)  0 (0.0%)  5 (2.4%)  10 (4.8%)  12 (5.8%)  20 (9.6%)  15 (7.2%)  35 (16.8%)  44 (21.2%)  30 (14.4%)  34 (16.3%)  7.2 (2.2)  3 (1.6%) | 2 (0.3%)  5 (0.7%)  -  21 (3.0%)  27 (3.8%)  50 (7.1%)  67 (9.5%)  114 (16.1%)  186 (26.3%)  129 (18.3%)  79 (11.2%)  7.5 (1.9)  26 (3.7%) | 2 (0.3%)  5 (0.8%)  -  17 (2.8%)  23 (3.8%)  41 (6.8%)  58 (9.6%)  96 (15.9%)  166 (27.5%)  104 (17.2%)  67 (11.1%)  7.4 (1.9)  24 (4.0%) | U = 76853, z = -1.08, p < 0.281 | U = 57158, z = -0.80, p < 0.425 |
| How would you rate your quality of life in the last two weeks? ^6^ | Very poor  Poor  Neither poor nor good  Good  Very good  Missing | 4 (1.7%)  29 (12.0%)  67 (27.7%)  101 (41.7%)  39 (16.1%)  2 (0.8%) | 4 (1.9%)  27 (13.0%)  62 (29.8%)  81 (38.9%)  32 (15.4%)  2 (1.0%) | 7 (1.0%)  46 (6.5%)  197 (27.9%)  330 (46.7%)  100 (14.2%)  26 (3.7%) | 7 (1.2%)  41 (6.8%)  179 (29.7%)  277 (45.9%)  74 (12.3%)  25 (4.1%) | *X^2^* (4) = 8.68, p = 0.070 | *X^2^* (4) = 10.25, p = 0.036 |

*# Question taken from IDEAL survey.*

Supplementary Table 5. Carers’ experience of caregiving

| Item | Response key | Participants | | Benchmarking data (where applicable) | | Statistical comparison  (where applicable) | |
| --- | --- | --- | --- | --- | --- | --- | --- |
|  |  | INCLUDE All carers  N(%) or mean(SD) | INCLUDE spouse/partners only  N(%) or mean(SD) | IDEAL T3 all carers N(%) or mean(SD) | IDEAL T3 spouse/partners only N(%) or mean(SD) | INCLUDE all carer vs benchmark | INCLUDE spouse/partners only vs benchmark |
| **Competence:** |  |  |  |  |  |  |  |
| How often do you feel confident that you are meeting the needs of [person with dementia]? ^7^ | Never  Some of the time  Most of the time  All of the time  Missing | 0 (0.0%)  42 (17.4%)  158 (65.3%)  41 (16.9%)  1 (0.4%) | 0 (0.0%)  35 (16.8%)  137 (65.9%)  35 (16.8%)  1 (0.5%) | 2 (0.3%)  108 (15.3%)  463 (65.6%)  104 (14.7%)  29 (4.1%) | 2 (0.3%)  83 (13.8%)  400 (66.3%)  90 (14.9%)  28 (4.6%) | *X^2^* (3) = 1.47, p = 0.690 | *X^2^* (3) = 1.74, p = 0.628 |
| How often do you feel that you are doing a good job as a carer? ^7^ | Never  Some of the time  Most of the time  All of the time  Missing | 1 (0.4%)  56 (23.1%)  141 (58.3%)  41 (16.9%)  3 (1.2%) | 0 (0.0%)  49 (23.6%)  119 (57.2%)  37 (17.8%)  3 (1.4%) | 9 (1.3.%)  128 (18.1%)  443 (62.7%)  101 (14.3%)  25 (3.5%) | 7 (1.2%)  101 (16.7%)  381 (63.2%)  91 (15.1%)  23 (3.8%) | *X^2^* (3) = 4.87, p = 0.182 | *X^2^* (3) = 7.72, p = 0.052 |
| How often do you feel competent in your ability to care for [person with dementia]? ^7^ | Never  Some of the time  Most of the time  All of the time  Missing | 1 (0.4%)  36 (14.9%)  136 (56.2%)  66 (27.3%)  3 (1.2%) | 0 (0.0%)  29 (13.9%)  118 (56.7%)  58 (27.9%)  3 (1.4%) | 4 (0.6%)  109 (15.4%)  432 (61.2%)  136 (19.3%)  25 (3.5%) | 2 (0.3%)  89 (14.8%)  371 (61.5%)  118 (19.6%)  23 (3.8%) | *X^2^* (3) = 6.30, p = 0.098 | *X^2^* (3) = 6.12, p = 0.106 |
| **Relationship with person with dementia:** |  |  |  |  |  |  |  |
| Generally how well do you and [the person with dementia] get along together? ^8^ | Not well at all  Not too well  Quite well  Well  Very well  Extremely well  Missing | 1 (0.4%)  14 (5.8%)  23 (9.5%)  34 (14.0%)  96 (39.7%)  73 (30.2%)  1 (0.4%) | 1 (0.5%)  13 (6.3%)  21 (10.1%)  32 (15.4%)  84 (40.4%)  56 (26.9%)  1 (0.5%) | 4 (0.6%)  18 (2.5%)  72 (10.2%)  121 (17.1%)  287 (40.7%)  172 (24.4%)  32 (4.2%) | 3 (0.5%)  14 (2.3%)  63 (10.4%)  96 (15.9%)  245 (40.6%)  151 (25.0%)  31 (5.1%) | *X^2^* (5) = 8.70, p = 0.122 | *X^2^* (5) = 6.92, p = 0.227 |
| **Coping:** |  |  |  |  |  |  |  |
| Do you feel you cope well as a carer? ^9^ | Never  Sometimes  Often  Always  Missing | 2 (0.8%)  67 (27.7%)  112 (46.3%)  58 (24.0%)  3 (1.2%) | 1 (0.5%)  59 (28.4%)  96 (46.2%)  49 (23.6%)  3 (1.4%) | 9 (1.3%)  220 (31.2%)  338 (47.9%)  112 (15.9%)  27 (3.8%) | 8 (1.3%)  181 (30.0%)  291 (48.3%)  98 (16.3%)  25 (4.1%) | *X^2^* (3) = 7.51, p = 0.057 | *X^2^* (3) = 5.64, p = 0.130 |
| **Social Restrictions:** |  |  |  |  |  |  |  |
| If you were ill, is there anybody who would step in to help [the person with dementia]? ^10^ | Yes, I could find someone quite easily  Yes, I could find someone but with some difficulty  No, there is no-one  Missing | 121 (50.0%)  77 (31.8%)  40 (16.5%)  4 (1.7%) | 99 (47.6%)  67 (32.2%)  38 (18.3%)  4 (1.9%) | 250 (35.4%)  298 (42.2%)  129 (18.3%)  29 (4.1%) | 212 (35.2%)  257 (42.6%)  107 (17.7%)  27 (4.5%) | *X^2^* (2) = 14.74, p = 0.001 | *X^2^* (2) = 10.22, p = 0.006 |
| If you needed a break from your caring role, is there someone who would look after [the person with dementia] for you? ^10^ | Yes, I could find someone quite easily  Yes, I could find someone but with some difficulty  No, there is no-one  Missing | 99 (40.9%)  79 (32.6%)  57 (23.6%)  7 (2.69%) | 81 (38.9%)  68 (32.7%)  52 (25.0%)  7 (3.4%) | 199 (28.2%)  275 (39.0%)  199 (28.2%)  33 (4.7%) | 162 (26.9%)  234 (38.8%)  176 (29.2%)  31 (5.1%) | *X^2^* (2) = 12.46, p = 0.002 | *X^2^* (2) = 9.90, p = 0.007 |
| **Role captivity:** |  |  |  |  |  |  |  |
| How much do you wish you were free to lead a life of your own? ^11^ | Not at all  Just a little  Somewhat  Very much  Missing | 69 (28.5%)  83 (34.3%)  46 (19.0%)  42 (17.4%)  2 (0.8%) | 61 (29.3%)  70 (33.7%)  38 (18.3%)  37 (17.8%)  2 (1.0%) | 198 (28.0%)  245 (34.7%)  152 (21.5%)  81 (11.5%)  30 (4.2%) | 182 (30.2%)  204 (33.8%)  122 (20.2%)  68 (11.3%)  27 (4.5%) | *X^2^* (3) = 5.07, p = 0.167 | *X^2^* (3) = 5.12, p = 0.163 |
| How much do you feel trapped by the person’s dementia? ^11^ | Not at all  Just a little  Somewhat  Very much  Missing | 65 (26.9%)  74 (30.6%)  44 (18.2%)  58 (24.0%)  1 (0.4%) | 54 (26.0%)  63 (30.3%)  37 (17.8%)  53 (25.5%)  1 (0.5%) | 168 (23.8%)  249 (35.3%)  162 (22.9%)  100 (14.2%)  27 (3.8%) | 138 (22.9%)  214 (35.5%)  142 (23.5%)  84 (13.9%)  25 (4.1%) | *X^2^* (3) = 13.68, p = 0.003 | *X^2^* (3) = 15.88, p = 0.001 |
| How much do you wish you could just run away? ^11^ | Not at all  Just a little  Somewhat  Very much  Missing | 127 (52.5%)  56 (23.1%)  40 (16.5%)  18 (7.4%)  1 (0.4%) | 107 (51.4%)  53 (25.5%)  31 (14.9%)  16 (7.7%)  1 (0.5%) | 398 (56.4%)  158 (22.4%)  83 (11.8%)  41 (5.8%)  26 (3.7%) | 343 (56.9%)  134 (22.2%)  73 (12.1%)  29 (4.8%)  24 (4.0%) | *X^2^* (3) = 4.21, p = 0.240 | *X^2^* (3) = 4.52, p = 0.210 |

Supplementary Table 6. Sensitivity analysis for statistically significant tests from Supplementary Tables 2-5 (all carers). The original statistical tests assume independence of samples when in reality the samples are partially overlapping. For these measures, overlapping samples t-tests for comparisons of means, or partially overlapping samples z-tests for comparisons of proportions are shown, as well as paired samples only tests.

| Measure | Original statistical test:  Chi-square test of independence or Mann-Whitney U Test | Overlapping samples tests:  Partially overlapping samples t-test (comparison of means) or Partially overlapping samples z-test (comparison of proportions) | Paired samples tests:  McNemar’s Chi-squared test or Friedman test |
| --- | --- | --- | --- |
| Considering the people to whom you are related either by birth, marriage, or adoption, how many relatives do you see or hear from at least once a month? | U = 95866, z = -3.99, p < 0.001 | t = 3.26, p = 0.001 | Friedman test (n = 150):  X^2^ (15) = 61.82, p < 0.001 |
| Do you feel lonely? | *X^2^* (2) = 55.62, p < 0.001 | t = -4.69, p < 0.001 | McNemar’s test (n = 150):  X^2^ (3), = 27.05, p < 0.001 |
| Overall, would you say you expect more good things to happen to you than bad? | *X^2^* (2) = 70.79, p < 0.001 | t = 0.11, p = 0.912 | Friedman test (n = 148):  X^2^ (4) = 13.86, p = 0.008 |
| On a scale of zero to 10, where zero is “not at all” and 10 is “very”, how satisfied are you with your life nowadays? | U = 73098, z = -2.65, p < 0.008 | t = -2.88, p = 0.004 | Friedman test (n = 152):  X^2^ (8) = 18.66, p = 0.017 |
| If you were ill, is there anybody who would step in to help [the person with dementia]? | *X^2^* (2) = 14.74, p = 0.001 | t = -3.21, p = 0.001 | McNemar’s test (n = 146):  X^2^ (3), = 11.36, p = 0.010 |
| If you needed a break from your caring role, is there someone who would look after [the person with dementia] for you? | *X^2^* (2) = 12.46, p = 0.002 | t = -3.35, p = 0.001 | McNemar’s test (n = 145):  X^2^ (3), = 14.00, p = 0.003 |
| How much do you feel trapped by the person’s dementia? | *X^2^* (3) = 13.68, p = 0.003 | t = 1.56, p = 0.118 | Friedman test (n = 151):  X^2^ (3), = 2.7, p = 0.440 |

Supplementary Table 7. Sensitivity analysis for statistically significant tests from Supplementary Tables 2-5 (spouse/partners only). The original statistical tests assume independence of samples when in reality the samples are partially overlapping. For these measures, overlapping samples t-tests for comparisons of means, or partially overlapping samples z-tests for comparisons of proportions are shown, as well as paired samples only tests.

| Measure | Original statistical test  Chi-square test of independence or Mann-Whitney U Test | Overlapping samples tests:  Partially overlapping samples t-test (comparison of means) or Partially overlapping samples z-test (comparison of proportions) | Paired samples tests:  McNemar’s Chi-squared test or Friedman test |
| --- | --- | --- | --- |
| Considering the people to whom you are related either by birth, marriage, or adoption, how many relatives do you see or hear from at least once a month? | U = 68494, z = -3.16, p = 0.002 | t = 2.50, p = 0.013 | Friedman test (n = 132):  X^2^ (14) = 58.34, p < 0.001 |
| Do you feel lonely? | *X^2^* (2) = 46.44, p < 0.001 | t = -3.85, p < 0.001 | McNemar’s test (n = 132):  X^2^ (3), = 19.08, p < 0.001 |
| Overall, would you say you expect more good things to happen to you than bad? | *X^2^* (4) = 37.32, p < 0.001 | t = -0.38, p = 0.702 | Friedman test (n = 134):  X^2^ (4) = 14.16, p = 0.007 |
| On a scale of zero to 10, where zero is “not at all” and 10 is “very”, how satisfied are you with your life nowadays? | U = 53043, z = -2.61, p = 0.009 | t = -2.84, p = 0.005 | Friedman test (n = 134):  X^2^ (8) = 20.18, p = 0.010 |
| How would you rate your quality of life in the last two weeks? | *X^2^* (4) = 10.25, p = 0.036 | t = -1.59, p = 0.113 | Friedman test (n = 133):  X^2^ (3) = 7.00, p = 0.072 |
| If you were ill, is there anybody who would step in to help [the person with dementia]? | *X^2^* (2) = 10.22, p = 0.006 | t = -2.14, p = 0.032 | McNemar’s test (n = 131):  X^2^ (3) = 9.52, p = 0.023 |
| If you needed a break from your caring role, is there someone who would look after [the person with dementia] for you? | *X^2^* (2) = 9.90, p = 0.007 | t = -2.92, p = 0.004 | McNemar’s test (n = 127):  X^2^ (3) = 12.23, p = 0.007 |
| How much do you feel trapped by [the person’s] dementia? | *X^2^* (3) = 15.88, p = 0.001 | t = 1.78, p = 0.076 | McNemar’s test (n = 133):  X^2^ (6), = 8.63, p = 0.196 |

**Supplementary 8: References for supplementary tables**

1. Bowling A. Just one question: If one question works, why ask several? *J Epidemiol Community Health.* 2005;59(5):342-345. 10.1136/jech.2004.021204

2. Lubben J, Blozik E, Gillmann G, et al. Performance of an abbreviated version of the Lubben Social Network Scale among three European community-dwelling older adult populations. *Gerontologist.* 2006;46(4):503-513. 10.1093/geront/46.4.503

3. Bech P. Measuring the dimension of psychological general well-being by the WHO-5. *Quality of Life Newsletter.* 2004;32:15-16.

4. Scheier MF, Carver CS, Bridges MW. Distinguishing optimism from neuroticism (and trait anxiety, self-mastery, and self-esteem): a reevaluation of the Life Orientation Test. *J Pers Soc Psychol.* 1994;67(6):1063-1078. 10.1037//0022-3514.67.6.1063

5. Office for National Statistics. Surveys using the 4 Office for National Statistics personal well-being questions. 2018; <https://www.ons.gov.uk/peoplepopulationandcommunity/wellbeing/methodologies/surveysusingthe4officefornationalstatisticspersonalwellbeingquestions>. Accessed 18/01/2018.

6. Skevington SM, Lotfy M, O'Connell KA, Group W. The World Health Organization's WHOQOL-BREF quality of life assessment: psychometric properties and results of the international field trial. A report from the WHOQOL group. *Qual Life Res.* 2004;13(2):299-310. 10.1023/B:QURE.0000018486.91360.00

7. Robertson SM, Zarit SH, Duncan LG, Rovine MJ, Femia EE. Family caregivers’ patterns of positive and negative affect. *Family Relations.* 2007;56(1):12-23.

8. Bengtson VL, Schrader SS. Parent-child relations. In: Mangon DJ, Peterson WA, eds. *Research instruments in social gerontology: Social roles and social participation.* Vol 2. Minnesota: University of Minnesota Press; 1982:115-185.

9. McKee K, Philp I, Lamura G, et al. The COPE index--a first stage assessment of negative impact, positive value and quality of support of caregiving in informal carers of older people. *Aging Ment Health.* 2003;7(1):39-52. 10.1080/1360786021000006956

10. Balducci C, Mnich E, McKee KJ, et al. Negative impact and positive value in caregiving: validation of the COPE index in a six-country sample of carers. *Gerontologist.* 2008;48(3):276-286. 10.1093/geront/48.3.276

11. Pearlin LI, Mullan JT, Semple SJ, Skaff MM. Caregiving and the stress process: An overview of concepts and their measures. *Gerontologist.* 1990;30(5):583-594.
